# Self-made thoracic needled suspending device with a snare: An excellent aid for uniportal video-assisted thoracic lobectomy and segmentectomy for lung cancer

**DOI:** 10.3892/ol.2020.11409

**Published:** 2020-02-18

**Authors:** Shanlei Wang, Chuizheng Meng, Zhongmin Jiang, Diego Gonzalez-Rivas, Junfen Ruan, Wei Xu, Chuanping Liu, Lei Zhang, Guogang Gao, Ge Yu, Hezhi Teng, Jin Ju

Oncol Lett 17: 3671-3676, 2019; DOI: 10.3892/ol.2019.10030

Subsequently to the publication of this article, the authors have realized that the details of the first listed address were presented incorrectly: “Mount Qianfuo Attatched Hospital of Shandong University” should have been written as “Shandong Provincial Qianfoshan Hospital, Shandong University”. Therefore, the correct author affliations for this article should have been presented as follows:

SHANLEI WANG^1,2^, CHUIZHENG MENG^2^, ZHONGMIN JIANG^1^. DIEGO GONZALEZ-RIVAS^3^, JUNFEN RUAN^2^, WEI XU^2^, CHUANPING LIU^2^, LEI ZHANG^2^, GUOGANG GAO^2^, GE YU^2^, HEZHI TENG^2^ and JIN JU^2^

^1^Department of Thoracic Surgery, **Shandong Provincial Qianfoshan Hospital, Shandong University**, Jinan, Shandong 250000; ^2^Department of Thoracic Surgery, Weihai Municipal Hospital, Weihai, Shandong 264200, P.R. China; ^3^Department of Thoracic Surgery (Coruña University Hospital) and Minimally Invasive Thoracic Surgery Unit (UCTMI), 15008 Coruña, Spain

The authors apologize for this oversight on their part, and for any inconvenience caused.

